# Tuning the size of a redox-active tetrathiafulvalene-based self-assembled ring

**DOI:** 10.3762/bjoc.11.108

**Published:** 2015-06-05

**Authors:** Sébastien Bivaud, Sébastien Goeb, Vincent Croué, Magali Allain, Flavia Pop, Marc Sallé

**Affiliations:** 1 Laboratoire MOLTECH-Anjou, Université d’Angers, UMR CNRS 6200, 2 bd Lavoisier, 49045 Angers Cedex, France. Fax: 0033241735439, Tel: 0033241735405

**Keywords:** coordination, metal-driven, redox, self-assembly, tetrathiafulvalene

## Abstract

The synthesis of a new Pd coordination-driven self-assembled ring M_6_L_3_ constructed from a concave tetrapyridyl π-extended tetrathiafulvalene ligand (exTTF) is described. The same ligand is also able to self-assemble in a M_4_L_2_ mode as previously described. Herein, we demonstrate that the bulkiness of the ancillary groups in the Pd complex allows for modulating the size and the shape of the resulting discrete self-assembly, which therefore incorporate two (M_4_L_2_) or three (M_6_L_3_) electroactive exTTF sidewalls.

## Findings

The coordination-driven approach is a well-established method that has been extensively used to reach more and more sophisticated cage-like discrete molecules [1–19], including redox-active ones [20]. In this context and since this strategy results from one single chemical step (metal to ligand assembly), there is a great interest in controlling the parameters which govern the final size and geometry of the resulting discrete self-assembled structures. Some general trends have first to be considered: i) as awaited from a lower kinetic stability, the ligand exchange process in the case of square-planar Pd(II) complexes is faster than with Pt(II) analogues; ii) the most thermodynamically stable species is formed along the assembly process, but if no evident energetic advantage exists for one structure, a dynamic equilibrium between two or more macrocyclic entities may be observed in solution [21–26]. This is in particular the case with flexible (including long) linear ditopic ligands, which favor the formation of triangle species whereas shorter ones shift the equilibrium towards molecular squares for which the enthalpic gain (less steric constraint) compensates for the entropic penalty. Beside the conformational flexibility of the ligand, parameters such as temperature, concentration and solvent type can influence the equilibrium. Isolation of the species from a given equilibrium has not been often carried out [27–28]. We were able in our case to operate the separation of a mixture of a triangle and a square [29]. The triangle–square dynamic equilibrium also depends on the nature of the ancillary ligand on the metal corner [21–22,26,30–33]. In particular, steric repulsions due to the ancillary ligand may displace the equilibrium towards the triangular species since the latter offers more space around the metal center. A change in the ancillary group can also lead to a modification of the cavity volume for a given cage [34]. Beyond those results, additional important issues still need to be addressed and concern in particular the possibility to obtain, from one given ligand, one single and stable assembly whose cavity size can be controlled.

We recently depicted the preparation and properties of redox-active rings [29,35] and cages [36–40] integrating the tetrathiafulvalene (TTF) skeleton. In particular, we described self-assembled containers prepared from an electron-rich ligand precursor based on the extended-TTF framework (exTTF) [39].

On this basis, we report herein that the size and the shape of coordination-driven self-assembled redox-active cages, constructed from a exTTF-based tetratopic ligand, can be tuned by modulating the bulkiness of the ancillary group on the metal complex precursor.

**Scheme 1:**
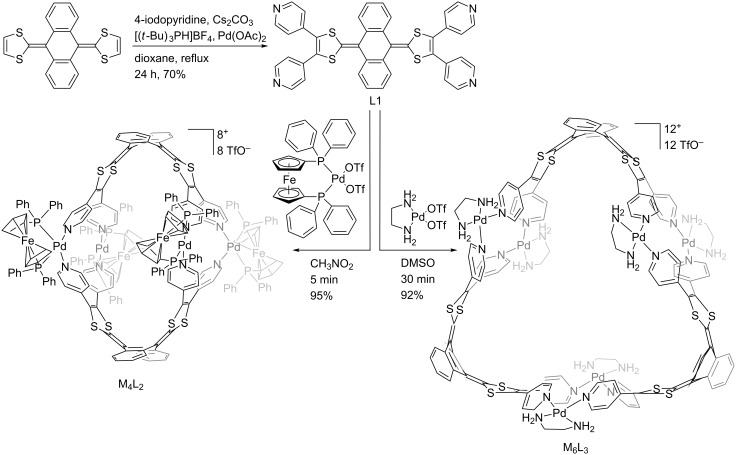
Synthesis of ligand L1, self-assemblies M_4_L_2_ and M_6_L_3_.

**Figure 1:**
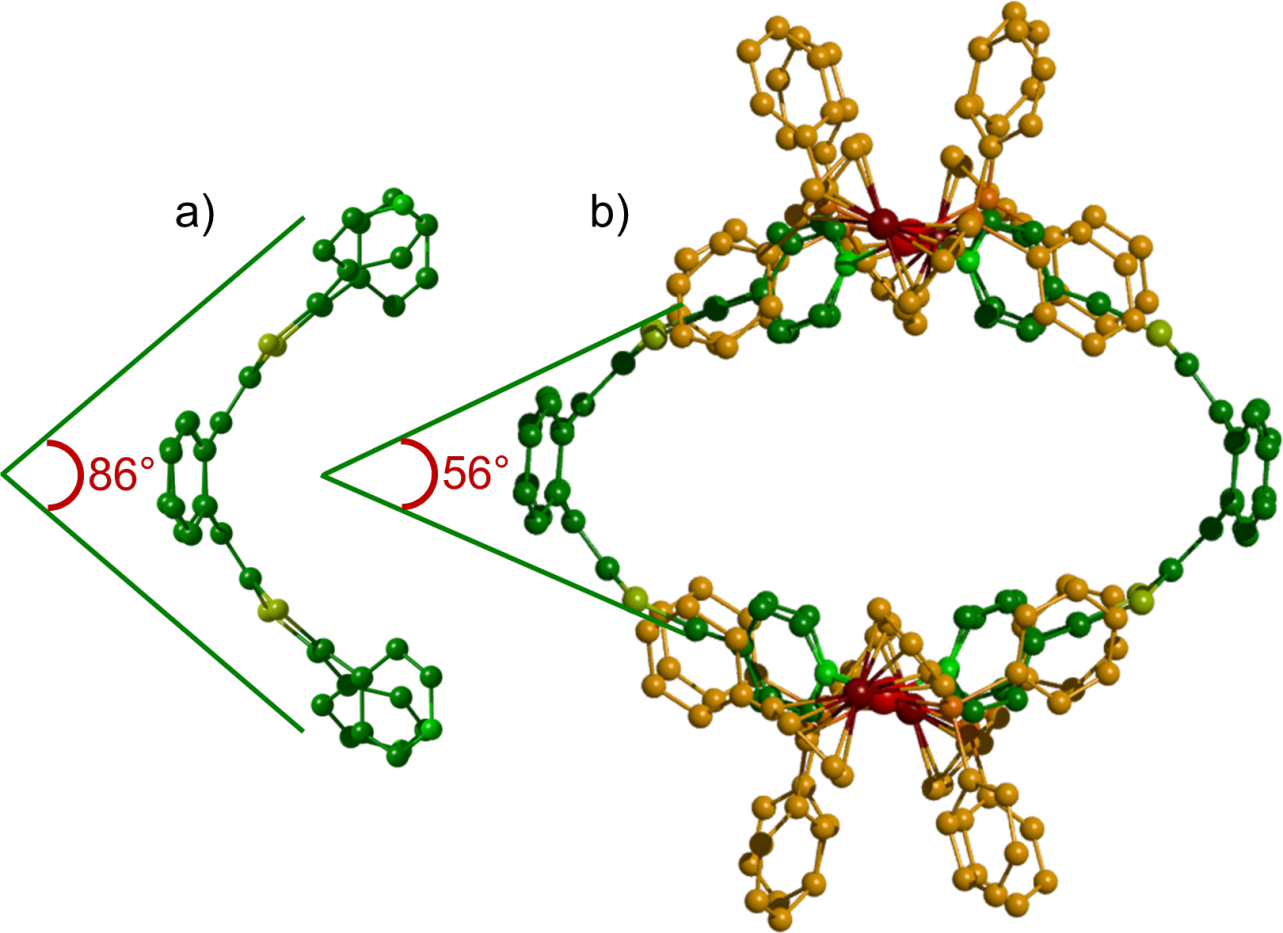
X-ray crystal structures of: (a) ligand L1, (b) self-assembly M_4_L_2_. For clarity, H atoms and TfO^−^ counteranions have been omitted.

The tetrapyridyl-exTTF ligand L1 (Scheme 1, Figure 1a) was synthesized through a palladium catalysed C–H arylation from the naked exTTF [39]. We already reported that the self-assembly process of this tetratopic ligand with *cis*-M(dppf)(OTf)_2_ (M = Pd or Pt; dppf = 1,1’-bis(diphenylphosphino)ferrocene; OTf = trifluoromethanesulfonate) in nitromethane at 40 °C converged into a single symmetrical M_4_L_2_ discrete species (Scheme 1, Figure 1b) [39]. It is worth noting that the through space interaction between the phenyl rings of the bulky 1,1’-bis(diphenylphosphino) ferrocene (dppf) coligand and the pyridine moieties force the exTTF unit to increase significantly its curvature in comparison to ligand L1 (56° vs 86° respectively between the 1,3-dithiol-2-ylidene mean planes (Figure 1)). This leads to the formation of the compact M_4_L_2_ assembly in which the pyridyl units are wedged between the dppf units, producing therefore a robust assembly affording an oblate spheroidal cavity. On this basis and considering the relative flexibility of the large exTTF moiety, we assumed that the bulkiness of the metal complex coligand could be adjusted to tune the macrocycle size and shape.

**Figure 2:**
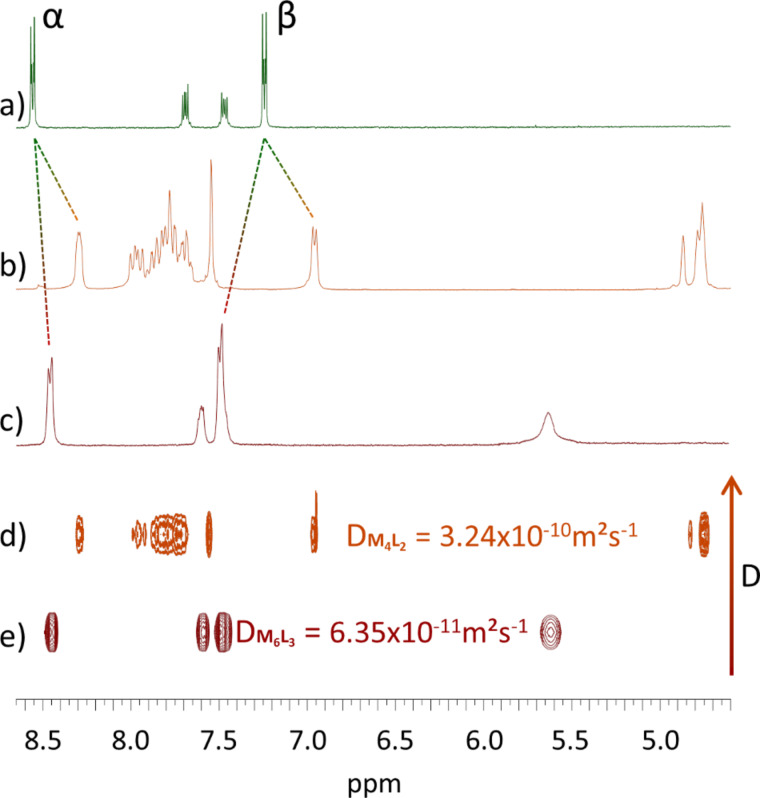
^1^H NMR, downfield region (α and β signals correspond respectively to α and β pyridyl protons): (a) L1 (DMSO-*d*_6_), (b) M_4_L_2_ (CD_3_NO_2_), (c) M_6_L_3_ (DMSO-*d*_6_), (d) DOSY NMR of M_4_L_2_ (CD_3_NO_2_) and (e) DOSY NMR of M_6_L_3_ (DMSO-*d*_6_).

Complexation of ligand L1 with precursor Pd(en)(OTf)_2_ (en = 1,2-ethylenediamine) was carried out in DMSO at 40 °C and monitored by ^1^H NMR. In 30 min, the reaction converged into a unique symmetrical discrete species that could be isolated in more than 90% yield after precipitation in ethyl acetate. In contrast with assembly M_4_L_2_ for which the presence of through-space interactions (Figure 4a) between the coligand phenyl units (dppf) and pyridyl groups result in an upfield shift of their signals (Figure 2b) compared to L1, the pyridyl protons are in this case shifted downfield (Figure 2c), as expected from coordination to a metal center. The corresponding DOSY NMR shows only one alignment of signals and confirms the formation of one unique species diffusing in solution with a D value of 6.35 × 10^−11^ m²·s^−1^ (Figure 2e). An estimated hydrodynamic radius (*R*_H_) of 17.2 Å could be calculated from the Stokes–Einstein equation (*T* = 298 K) for this new discrete system [41]. This result indicates that the latter is larger than the already described M_4_L_2_ container (*R*_H_ = 10.8 Å (Figure 2d)), and that the corresponding size is compatible with the formation of a M_6_L_3_ assembly (Scheme 1).

ESI–MS mass spectroscopy experiments were carried out in acetone and agree with a M_6_L_3_ stoichiometry in the gas phase for the new assembly, with multicharged isotopic patterns at *m*/*z* = 2278.3, 1468.9, 1064.4, 821.8, corresponding respectively to [M_6_L_3_-10TfO^−^]^2+^, [M_6_L_3_-9TfO^−^]^3+^, [M_6_L_3_-8TfO^−^]^4+^, [M_6_L_3_-7TfO^−^]^5+^ species and matching perfectly with theoretical ones (Supporting Information File 1, Figure S8).

**Figure 3:**
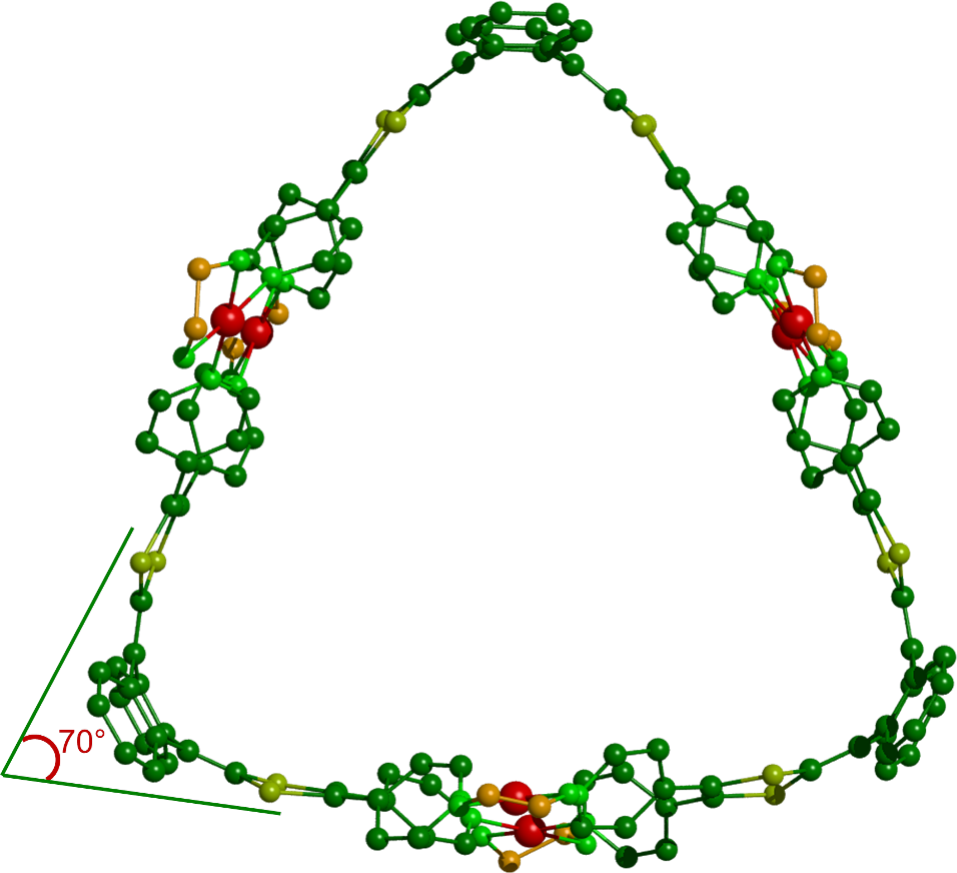
X-ray crystal structure of self-assembly M_6_L_3_. For clarity, H atoms and TfO^−^ counteranions have been omitted.

**Figure 4:**
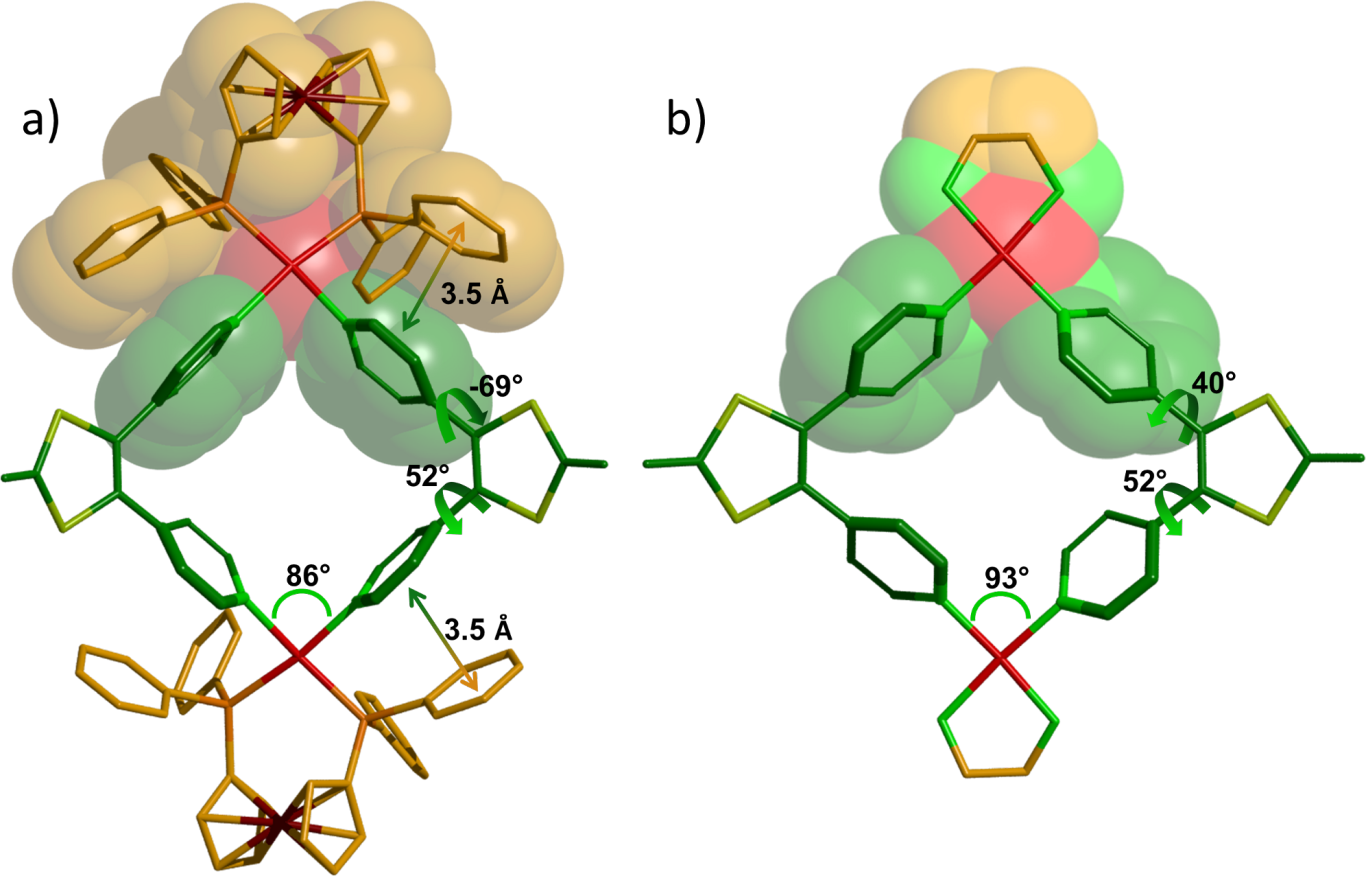
Geometry (from XRD) around two Pd centers in M_4_L_2_ (a) and M_6_L_3_ (b). The exTTF moieties have been cut for clarity.

Single crystals of assembly M_6_L_3_ were grown by slow diffusion of ethyl acetate in DMSO and allowed for determining unambiguously the solid-state structure by a synchrotron X-ray diffraction study (Figure 3). Whereas the sterically demanding dppf moiety leads to a M_4_L_2_ structure characterized by i) exTTF moieties which are highly distorted and ii) short interplanar distances between the phenyl units of the ancillary dppf complex and the pyridyl rings (3.5 Å) (Figure 4a), a much less constrained system is observed in the case of the M_6_L_3_ complex, characterizing a prismatic structure which is not driven by steric effects but which is mainly governed by thermodynamic aspects.

The M_6_L_3_ assembly forms a trigonal prismatic structure presenting a cavity defined by 17.7 Å high, 19.0 Å edge and 9.5 Å depth. The curvature of the exTTF moiety in the complex (70° between the 1,3-dithiol-2-ylidene mean plans, Figure 3) is intermediate between those observed for the free ligand L1 and the ligand in M_4_L_2_, which illustrates a lower ring constraint than in M_4_L_2_. In addition to the expected variation of the *N*(pyridyl)-Pd-*N*(pyridyl) angle within the distorted square planar – i.e. the dppf complex (M_4_L_2_, 86°) and the en one (M_6_L_3_, 93°) (Figure 4) –, the change in the ancillary ligand also results in a modification of the rotation angles between the pyridyl units and the 1,3-dithiol-2-ylidene heterocycles. Because of the lower steric demand with the 1,2-ethylenediamine co-ligand, the vicinal pyridyl units are free to rotate around the C-pyridine axis in M_6_L_3_, resulting in dihedral angles of 40° and 52° in the crystal (Figure 4b). These values are in the same range as those observed in the free ligand L1 (35° and 63°). By comparison, the pyridyl units in complex M_4_L_2_ are tilted with angles of 52° and −69° in the solid (Figure 4a). Those higher values result from the increased steric demand generated by the dppf coligand.

**Figure 5:**
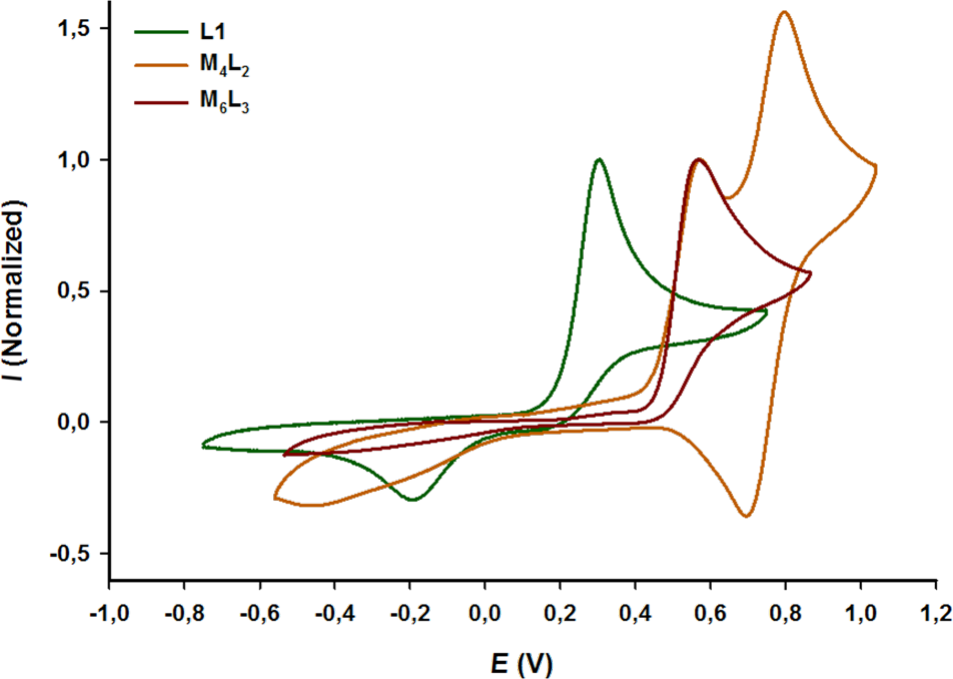
Cyclic voltammogram of L1 (*c* = 10^−3^ M, CH_3_CN/CH_2_Cl_2_ (v/v 50/50), 0.1 M *n*-Bu_4_NPF_6_, 100 mV·s^−1^, Pt), M_4_L_2_ and M_6_L_3_ (*c* = 10^−3^ M, CH_3_CN, 0.1 M *n-*Bu_4_NPF_6_, 100 mV·s^−1^, Cgr), V vs Fc/Fc^+^.

A cyclic voltammetry study of prism M_6_L_3_ was carried out in acetonitrile containing 0.1 M NBu_4_PF_6_ (Figure 5). Compared to ligand L1 which presents the usual electrochemical behavior of exTTF derivatives, i.e. one poorly electrochemically reversible two-electrons oxidation process, the oxidation of exTTF in self-assembly M_6_L_3_ is shifted to higher potential (+0.26 V), which is mainly attributed to the coordination to the Pd center. It can be noted that the redox behavior of M_6_L_3_ is very similar to the one of M_4_L_2_ [42], with an irreversible oxidation occurring exactly at the same potential (*E*_ox_ = +0.57 V vs Fc/Fc^+^), illustrating the fact that the nature of the ancillary ligand does not impact the electronic properties of exTTF in the corresponding self-assemblies.

In summary, two different coordination-driven discrete self-assemblies varying by the size and the shape can be built from the same tetratopic exTTF-based ligand, simply by changing the ancillary group on the Pd metal center. In particular, whereas a 1,1’-bis(diphenylphosphino)ferrocene co-ligand promotes a clipping of the ligand pyridyl units and leads to a strong curvature of the exTTF moiety integrated in a M_4_L_2_ coordination cage, the use of a smaller co-ligand leads to the formation of a larger M_6_L_3_ cavity in which the curvature of the exTTF is closer to ligand L1. The new M_6_L_3_ system has been fully characterized and exhibits electrochemical properties which are essentially similar to those of M_4_L_2_, indicating that the strong π-donating ability of the cavity can be maintained while enlarging its size, and illustrating the high potential of the coordination-driven approach in tuning the size and the shape of a target cavity. This approach constitutes a promising strategy to address the design of organic materials (e.g. for organic photovoltaics or molecular electronic devices). Indeed, mastering the geometry of multicomponent redox-active systems offers a unique opportunity to fine-tuning electronic interactions within the material [43], an issue which is of prime importance for optimizing electron transport in organic materials.

## Supporting Information

File 1 General methods, synthetic procedures, spectroscopic data.

File 2 X-ray crystallographic data CCDC 1043205.
