# Supplementary material for: Tuning the size of a redox-active tetrathiafulvalene-based self-assembled ring
Source: Beilstein J Org Chem. 2015 Jun 5;11:966–71. doi: 10.3762/bjoc.11.108 (PMC4464414; doi:10.3762/bjoc.11.108)
Supplement: File 1 — General methods, synthetic procedures, spectroscopic data. [file Beilstein_J_Org_Chem-11-966-s001.pdf]

Supporting Information

for

Tuning the size of a redox-active tetrathiafulvalene-based self-assembled ring

Sébastien Bivaud, Sébastien Goeb,* Vincent Croué, Magali Allain, Flavia Pop, Marc Sallé*

Address: Laboratoire MOLTECH-Anjou, Université d'Angers, UMR CNRS 6200,
2 bd Lavoisier, 49045 Angers Cedex, France. Fax: 0033241735439, Tel: 0033241735405

Email: Sébastien Bivaud - sebastien.goeb@univ-angers.fr; Marc Sallé - marc.salle@univ-angers.fr
*Corresponding author

General methods, synthetic procedures, spectroscopic data

CHEMICALS, INSTRUMENTATION AND TITRATION **S2**

Chemicals s2

Instrumentation s2

EXPERIMENTAL PROCEDURE AND CHARACTERIZATION DATA **S2**

NMR SPECTRA **S3**

Figure S1. ^{1}H NMR spectra of L1 in DMSO-d_6 s3

Figure S2. ^{1}H NMR spectra of M_4L_2 in CD_3NO_2 s3

Figure S3. DOSY NMR spectrum of M_4L_2 in CD_3NO_2 s4

Figure S4. ^{1}H NMR spectra of M_6L_3 in DMSO-d_6 s4

Figure S5. ^{19}F NMR spectrum of M_6L_3 in DMSO-d_6 s5

Figure S6. COSY NMR spectrum of M_6L_3 in DMSO-d_6 s5

Figure S7. DOSY NMR spectrum of M_6L_3 in DMSO-d_6 s6

MASS SPECTROMETRY ANALYSIS **S7**

Figure S7. HRMS Mass spectrometry of M_6L_3 s7

X-RAY STRUCTURES **S7**

Chemicals, instrumentation and titration

Chemicals

All reagents were of commercial reagent grade and were used without further purification. Ligand L1,[1] complex M_4L_2[1] and enPd(OTf)$_2$,[2] (en = ethylenediamine; OTf = trifluoromethane-sulfonate) were synthesized as described in literature.

Instrumentation

The 300.3 (^{1}H, DOSY, COSY), and 282.6 MHz (^{19}F) NMR spectra were recorded at room temperature using perdeuterated solvents as internal standards (^{1}H), external $CFCl_3$ (^{19}F), on a NMR Bruker Avance III 300 spectrometer. ESI-HRMS spectra were achieved on a Bruker Bruker MaXis 4G (upgrade of MicrO-Tof-Q 2) spectrometer in acetone (100 mmol). Cyclic voltammetry experiments were carried out on a BioLogic SP150 potentiostat, and the conditions were the following: 0.1 M *n*-Bu_4NPF_6 in acetonitrile or acetonitrile/dichloromethane, Ag/Ag^+ reference electrode, GC or Pt working electrode, and Pt counter electrode, 100 mV.s^{-1}, calibrated using internal ferrocen.
X-ray single-crystal diffraction data were collected at 120 K on the Cristal beamline at the SOLEIL Synchrotron (Saint-Aubin-France) on an Agilent 4-circle diffractometer equipped with an Atlas CCD detector. The experiment was carried out with radiation wavelength of 0.67 Å. The structure was solved by direct methods, expanded and refined on F^2 by full matrix least-squares techniques using SHELXS97 (G.M. Sheldrick, 1998) and SHEXL-2013 (G. M. Sheldrick 1993–2013, Version 2013/4) programs. All non-H atoms were refined anisotropically and empirical absorption was corrected by CrysAlisPro program (CrysAlisPro, Agilent Technologies, V1.171, 2012). The H atoms were included in the calculation without refinement.

Experimental procedure and characterization data

Complex M_6L_3

A mixture of ligand L1 (10.0 mg, 14.5 µmol) and enPd(OTf)$_2$ (13.5 mg, 29.0 µmol) in dimethyl sulfoxide (1 mL) was heated at 50°C for 30 min. Then, ethyl acetate (5 mL) was added and the mixture was centrifuged. The residue was washed with ethyl acetate (2 mL), diethyl ether (4 mL) and dried under vacuum to give complex M_6L_3 (21.6 mg, 36.3 µmol, 92%) as an orange solid. Monocrystals (orange, small lozenges) were obtained by slow diffusion of ethyl acetate in dimethyl sulfoxide (gaz–liquid). ^{1}H NMR (DMSO-d_6) δ 8.46 (d, ^3J = 5.9 Hz, 24H), 7.60 (m, 12H), 7.49 (m, 36H), 5.63 (brs, 24H), 2.65 (brs, 24H); ^{19}F NMR (DMSO-d_6) δ −76.88; ESI-HRMS m/z: 1468.8899 [M_6L_3-9TfO$^-$]$^{3+}$, 1064.4296 [M_6L_3-8TfO$^-$]$^{4+}$, 821.7533 [M_6L_3-9TfO$^-$]$^{5+}$; mp > 260 °C.

NMR spectra

Figure S1. ^{1}H NMR spectra of L1 in DMSO-d_6

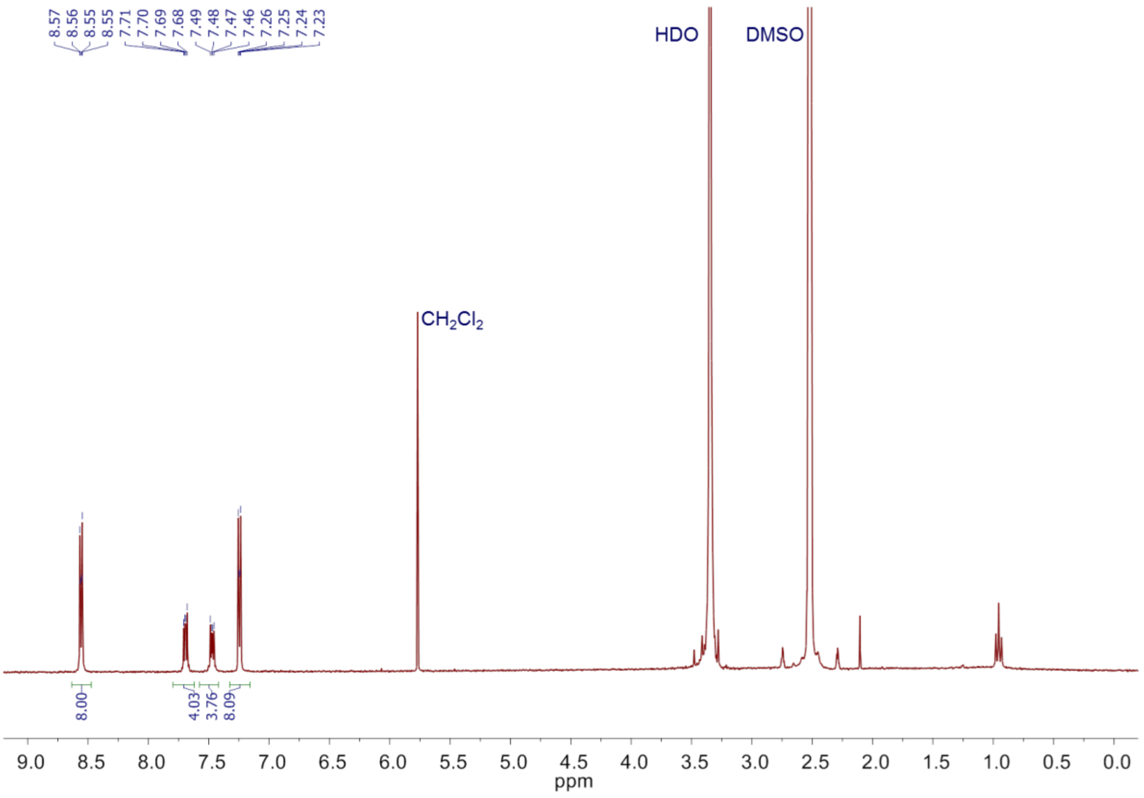

Figure S2. ^{1}H NMR spectra of M_4L_2 in CD_3NO_2

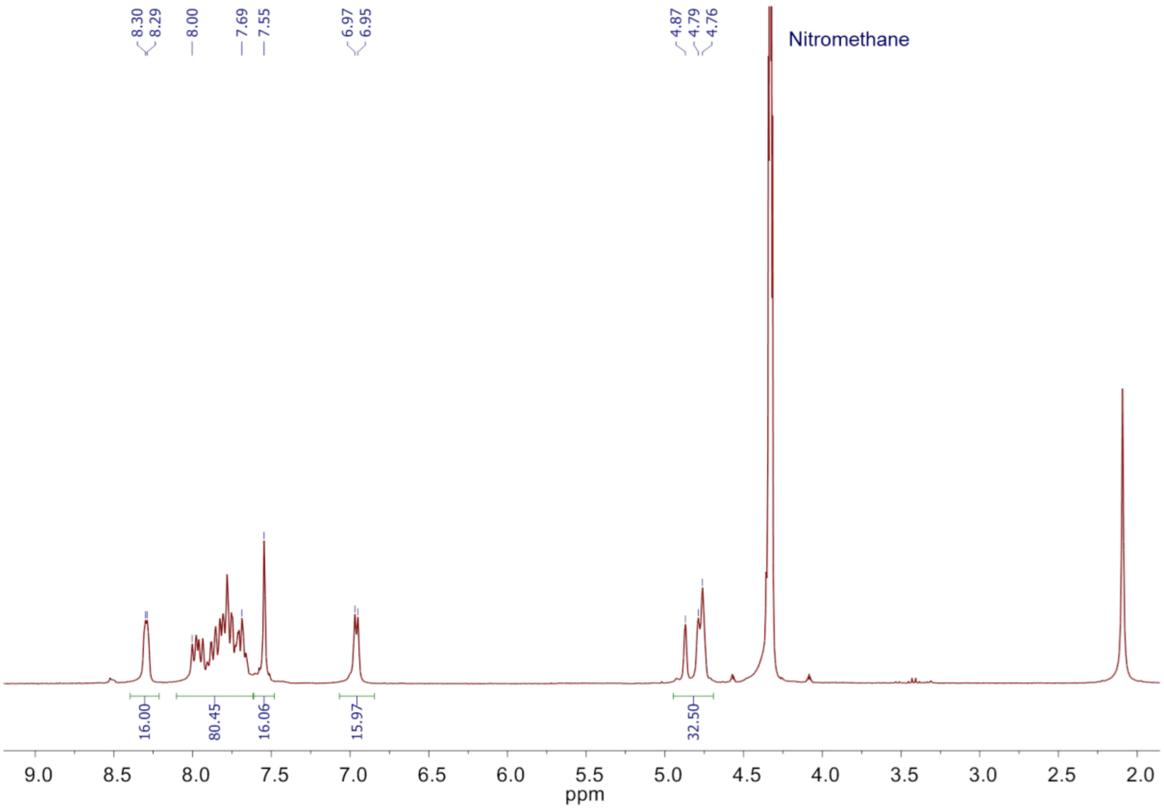

Figure S3. DOSY NMR spectrum of M_4L_2 in CD_3NO_2

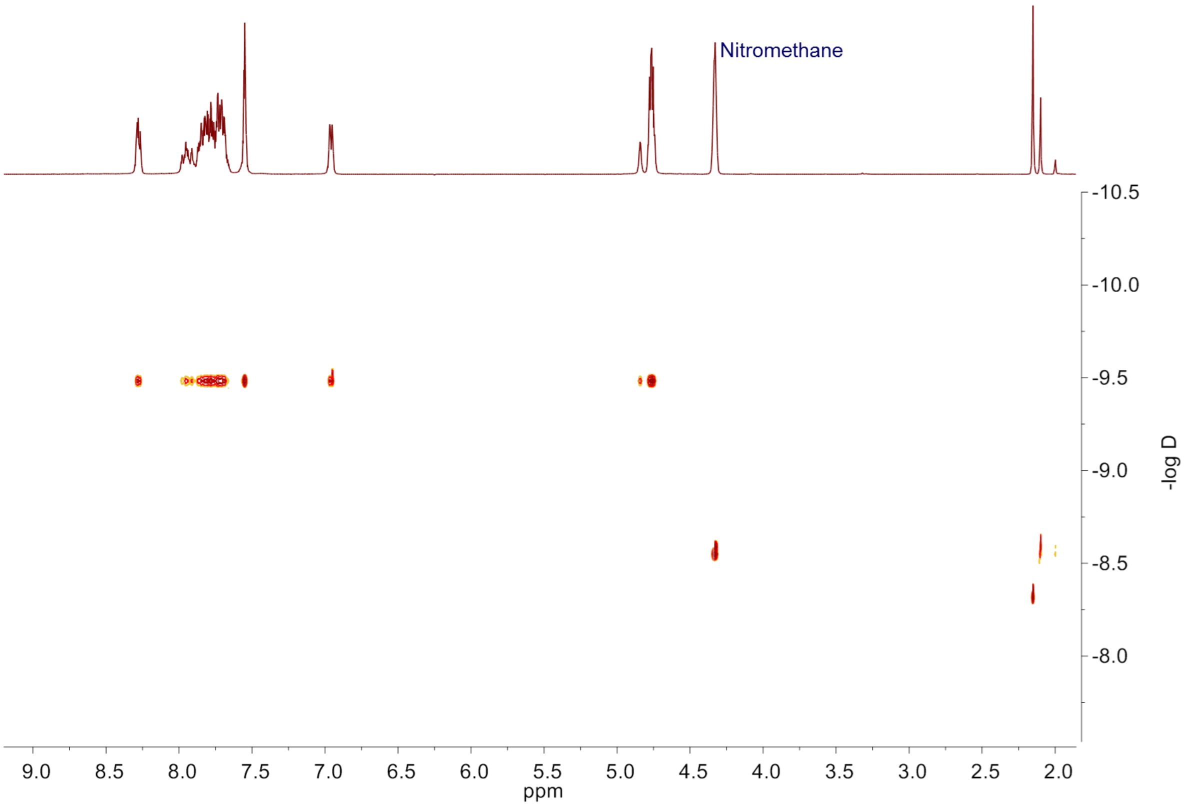

Figure S4. ^{1}H NMR spectra of M_6L_3 in DMSO-d_6

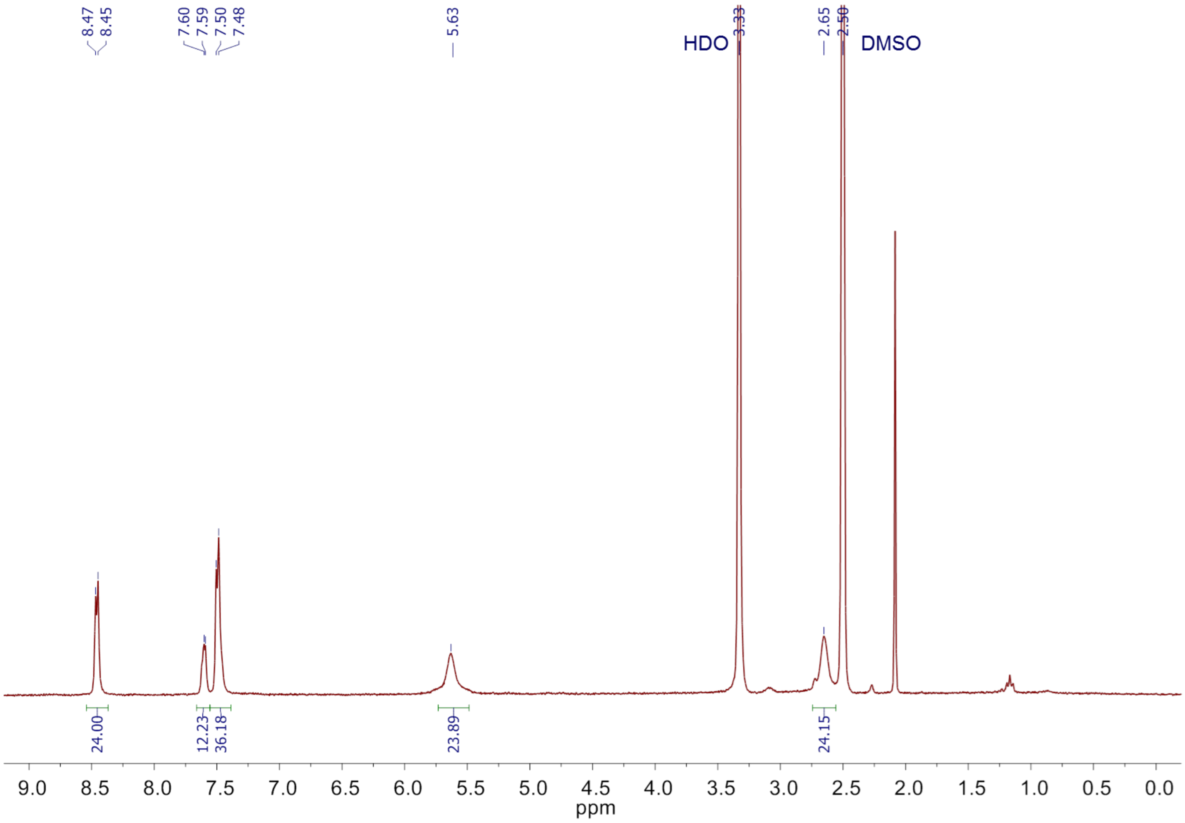

Figure S5. ^{19}F NMR spectrum of M_6L_3 in DMSO-d_6

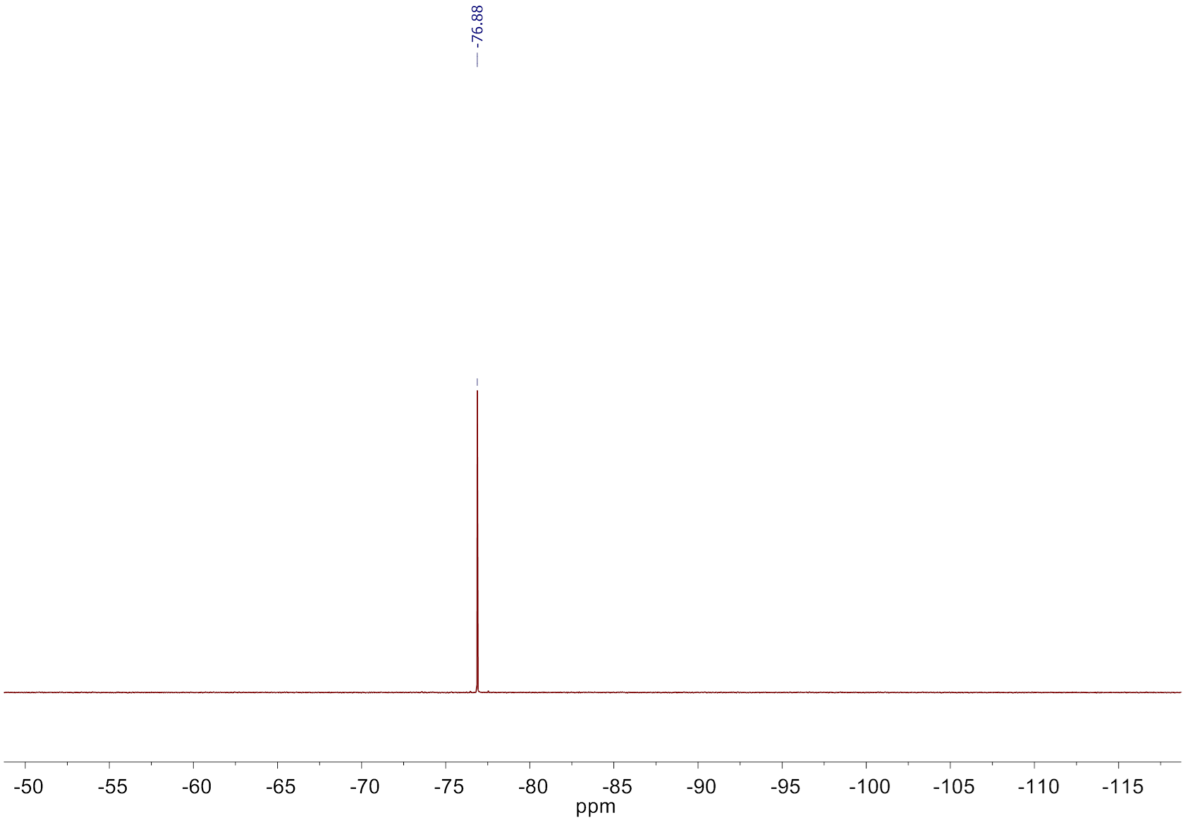

Figure S6. COSY NMR spectrum of M_6L_3 in DMSO-d_6

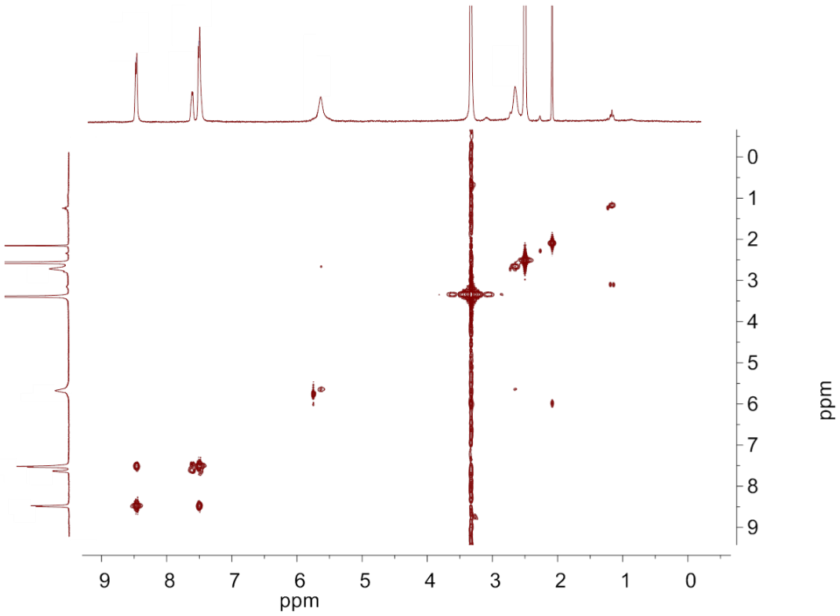

Figure S7. DOSY NMR spectrum of M_6L_3 in DMSO-d_6

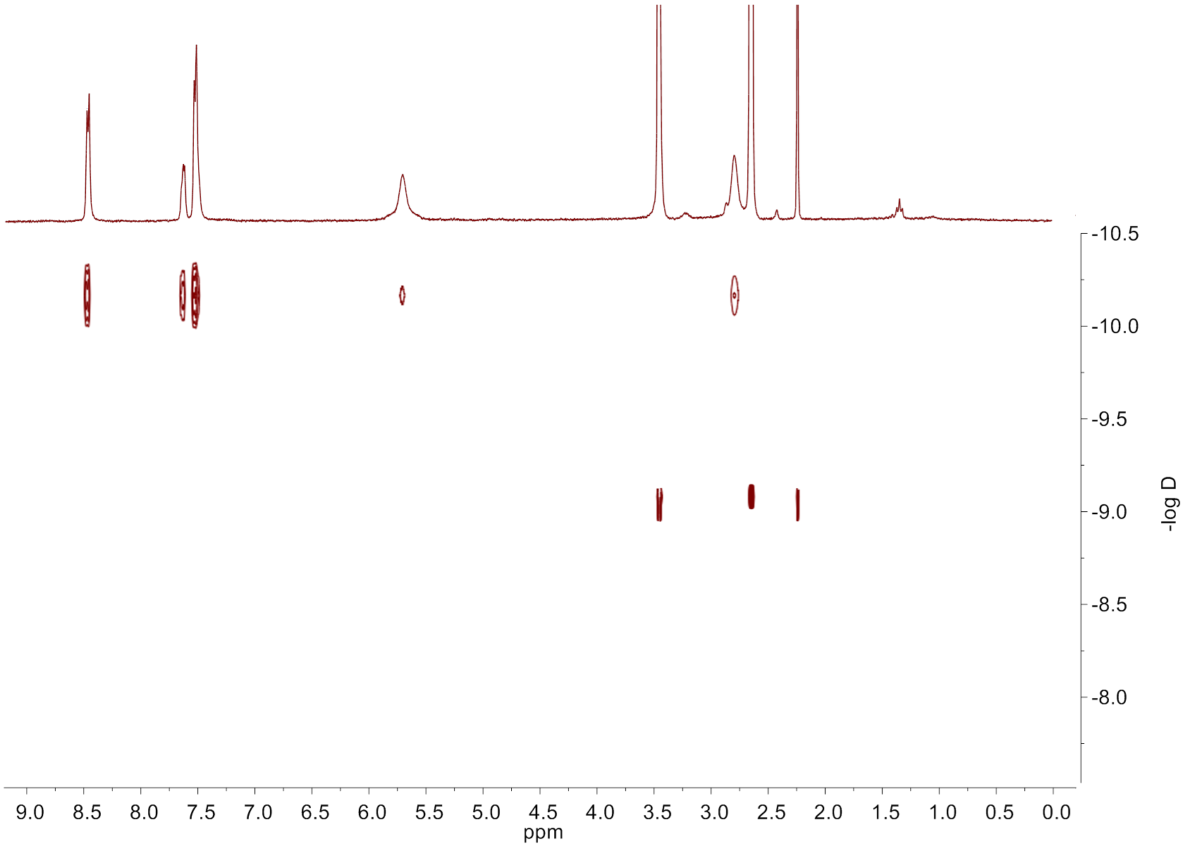

Mass spectrometry analysis

Figure S8. HRMS Mass spectrometry of M_6L_3

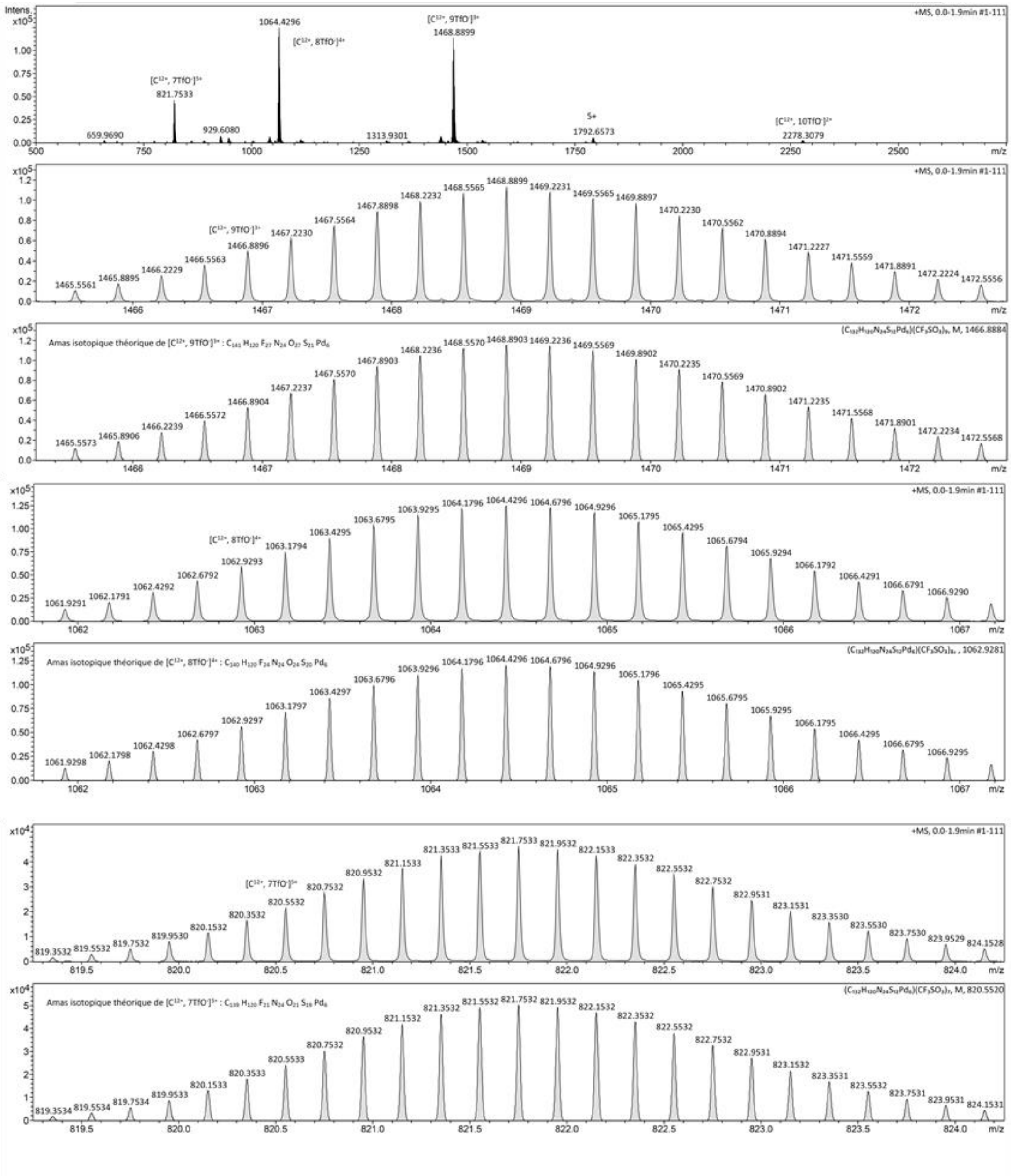

X-ray structures

Slow diffusion of ethyl acetate in a solution of M_6L_3 in DMSO afforded large orange crystals that tend to decompose rapidly. For this reason, diffraction data with low intensity at theta around 25° and thus low completeness could be collected. Nevertheless, we succeeded in solving the main structure and could determine 4 triflate anions. To improve the refinement, we used PLATON/SQUEEZE command. The unit cell contains anions and solvent molecules which were not modelled but the corresponding scattering contribution were taken into account using SQUEEZE/PLATON procedure. The unit cell contains 1 void of 20881 A^3 containing about 8104 electrons from which 2336 electrons can be attributed to the 32

missing CF_3SO_3 anions in the unit cell. The others 5768 electrons can be attributed to solvent molecules. As the solvent composition is not well known, it has not been included in the calculation of the empirical formula, formula weight, density, linear absorption coefficient and F(000).

Crystallographic data for M_6L_3: $C_{136}H_{120}F_{12}N_{24}O_{12}Pd_6S_{16}$, M = 3661.91, red prism, 0.19 x 0.11 x 0.05 mm^3, monoclinic, space group $P2_1/n$, a = 23.4015(2) Å, b = 34.9475(2) Å, c = 39.9599(4) Å, β = 91.391(1)°, V = 32670.5(5) $Å^3$, Z = 4, ρcalc = 0.744 g/cm^3, μ = 0.354 mm^{-1}, F(000) = 7360, θmin = 1.460°, θmax = 25.091°, 701627 reflections collected, 62714 unique (R_{int} = 0.11), parameters / restraints = 1855 / 1, R1 = 0.0917 and wR2 = 0.2636 using 49042 reflections with I>2σ(I), R1 = 0.1043 and wR2 = 0.2787 using all data, GOF = 1.057, $-1.057 < \Delta\rho < 2.823 e.Å^{-3}$.

References

[1] S. Bivaud, S. Goeb, V. Croue, P. I. Dron, M. Allain and M. Salle, *J. Am. Chem. Soc.*, 2013, **135**, 10018-10021.

[2] K. Tambara and G. D. Pantos, *Org Biomol Chem*, 2013, **11**, 2466-2472.
